# Immunmodulatory Treatment Strategies of Hepatocellular Carcinoma: From Checkpoint Inhibitors Now to an Integrated Approach in the Future

**DOI:** 10.3390/cancers13071558

**Published:** 2021-03-29

**Authors:** Matthias Ocker, Christian Mayr, Tobias Kiesslich, Sebastian Stintzing, Daniel Neureiter

**Affiliations:** 1 Department of Gastroenterology (Campus Benjamin Franklin), Charité University Medicine Berlin, 10117 Berlin, Germany; matthias.ocker@charite.de; 2 Translational Medicine & Clinical Pharmacology, Boehringer Ingelheim Pharma GmbH & Co. KG, 55216 Ingelheim, Germany; 3 Institute of Physiology and Pathophysiology, Paracelsus Medical University, 5020 Salzburg, Austria; christian.mayr@pmu.ac.at (C.M.); tobias.kiesslich@pmu.ac.at (T.K.); 4 Department of Internal Medicine I, Paracelsus Medical University, University Hospital Salzburg (SALK), 5020 Salzburg, Austria; 5 Division of Hematology, Oncology, and Tumor Immunology (Campus Charité Mitte), Medical Department, Charité University Medicine Berlin, 10117 Berlin, Germany; sebastian.stintzing@charite.de; 6 Institute of Pathology, Paracelsus Medical University, University Hospital Salzburg (SALK), 5020 Salzburg, Austria; 7 Cancer Cluster Salzburg, 5020 Salzburg, Austria

**Keywords:** hepatocellular carcinoma, immunotherapy, immune checkpoint inhibitors, locoregional treatment

## Abstract

**Simple Summary:**

Hepatocellular carcinoma (HCC) is among the most common cancer diseases worldwide and has only limited treatment options at advanced disease stages. Activation of the immune system with checkpoint inhibitors has revolutionized cancer medicine and has become important also for HCC treatment. Here, we summarize the current status of immunotherapy options for HCC and highlight how combination with locoregional therapies could improve the outcome of patients. Novel pathways and targets for immunologic drug development are briefly discussed that could help to increase the response rate of these approaches in HCC.

**Abstract:**

Background: Hepatocellular carcinoma (HCC) still represents a human tumor entity with very limited therapeutic options, especially for advanced stages. Here, immune checkpoint modulating drugs alone or in combination with local ablative techniques could open a new and attractive therapeutic “door” to improve outcome and response rate for patients with HCC. Methods: Published data on HCC experimental to pre-(clinical) treatment strategies from standard of care to novel immunomodulatory concepts were summarized and discussed in detail. Results: Overall, our knowledge of the role of immune checkpoints in HCC is dramatically increased in the last years. Experimental and pre-clinical findings could be translated to phase 1 and 2 clinical trials and became standard of care. Local ablative techniques of HCC could improve the effectivity of immune checkpoint inhibitors in situ. Conclusions: This review demonstrates the importance of immunomodulatory treatment strategies of HCC, whereby the “best treatment code” of immune checkpoint drugs, combination with ablative techniques and of timing must be evaluated in coming clinical trials.

## 1. Introduction

Liver cancer represents a considerable health issue due to an increasing incidence in most regions worldwide. It accounts for about 840,000 new cases and 780,000 estimated deaths–ranking 6th by incidence and 4th by cancer-related mortality for both sexes [1,2,3]. A clear male preponderance (2–3 times higher, up to five times in some countries [3,4]) is reflected by the age-standardized worldwide incidence rate of 13.9 and 4.9 per 100,000 male and female inhabitants, respectively [2]. Both, incidence and mortality rates vary by region mapping to the geographical distribution of viral hepatitis B/C (HBV/HCV) which are the most important causes of chronic liver disease and HCC [3,5]: while the highest numbers are found in eastern Asia with incidence/mortality rates of 17.7/16.0, respectively, Europe records about 4.0–6.8 new cases and 3.8–5.3 deaths from liver cancer and North America has about 6.6 new cases and 4.8 deaths per 100,000 inhabitants, for example [2]. These epidemiologic figures describe the situation for primary liver cancer which mainly compromises cases with hepatocellular carcinoma (HCC, 75–85%), besides 10–15% cases of intrahepatic cholangiocarcinoma as well as other rare tumors [1]. 

**Figure 1:**
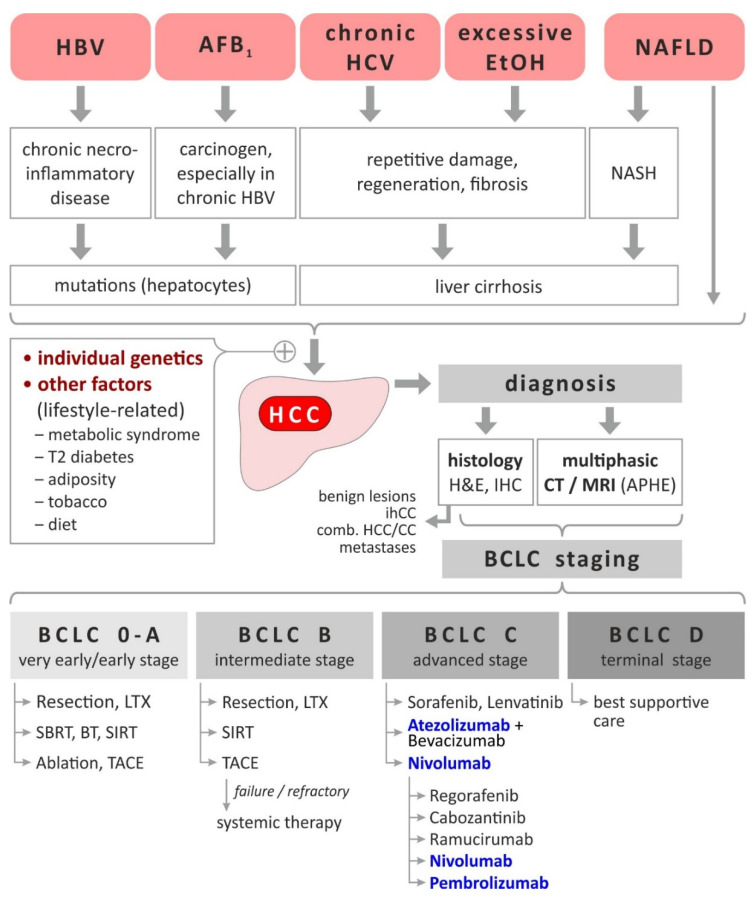
HCC-Etiology, risk factors, diagnosis and staging-dependent current treatment. Based on [3,5,8,15]. Immunomodulatory treatments are highlighted bold and blue. Abbreviations: AFB_1_, aflatoxin B_1_; APHE, arterial phase hyperenhancement; BCLC, Barcelona Clinic Liver Cancer; BT, brachytherapy; CT, computed tomography; EtOH, ethanol; H(B/C)V, hepatitis B/C virus; H & E, hematoxylin & eosin; HCC, hepatocellular carcinoma; (ih)CC), (intrahepatic) cholangiocarcinoma; IHC, immunohistochemistry; LTX, liver transplantation; MRI, magnetic resonance imaging; NAFLD, nonalcoholic fatty liver disease; NASH, nonalcoholic steatohepatitis; SBRT, stereotactic body radiotherapy; SIRT, selective internal radiotherapy; T2 diabetes, type 2 diabetes; TACE, transarterial chemoembolisation.

Figure 1 summarizes the main risk factors for development of HCC which include HBV, HCV, excessive alcohol consumption, metabolic syndrome, type-2 diabetes, obesity, non-alcoholic fatty liver disease (NAFLD), aflatoxin B_1_ (AFB_1_), tobacco, dietary factors (coffee decreases while high iron intake increases the HCC risk), as well as individual genetics (e.g., mutations in genes responsible for hemochromatosis, alpha-1-antitrypsin deficiency, glycogen storage disease, porphyrias, tyrosinemia, and Wilson’s disease) [3]. Accordingly, programs for prevention of HCC showed considerable efficiency, e.g., by a 80%/92% reduction of HCC incidence/mortality after neonatal HBV vaccination in Taiwan [6] and a 71% reduction of HCC risk by antiviral therapy achieving sustained virological response (SVR, [7]). 

As reviewed by others [5,8], surveillance for HCC is based on abdominal ultrasound and includes patients with liver cirrhosis, chronic HBV carriers or HCV-infected subjects with bridging fibrosis as well as patients with HCV infection and advanced fibrosis. Such surveillance might be supplemented in future by liquid biopsy [8,9,10] or other blood tests (e.g., GALAD score [11,12]). Currently, diagnosis of HCC is primarily based on imaging using computed tomography (CT) or magnetic resonance imaging (MRI) taking into account the typical vascular characteristics of HCC [5,13]. While the formal pathological proof is not mandatory for diagnosis of HCC, histopathological analyses by hematoxylin & eosin (H&E) supplemented by specific immunohistochemical analysis (IHC) allows for discrimination of HCC from benign or premalignant lesions (dysplastic nodules, hepatocellular adenoma, focal nodular hyperplasia) or intrahepatic cholangiocarcinoma (ihCC), combined HCC/CC and metastases of other primary tumors [5]. As summarized in Figure 1, confirmed cases of HCC undergo staging for optimal patient stratification and decision on subsequent therapeutic approaches: the most commonly used Barcelona Clinic Liver Cancer system (BCLC) integrates tumor stage, liver function parameters, cancer-related symptoms and performance status and classifies HCC patients into five categories (0, A–D) [14]. While very early or early stage HCC (BCLC 0, A) patients are eligible for curative surgical treatment (including liver transplantation) and locoregional ablation yielding survival times of >5 years, BCLC B (intermediate stage) patients currently receive transarterial chemoembolisation (TACE) associated with <2–5 years survival. Systemic treatment with multikinase inhibitors (e.g., sorafenib) in patients at BCLC stage C (advanced) usually achieves survival times of up to one year [14]. 

**Figure 2:**
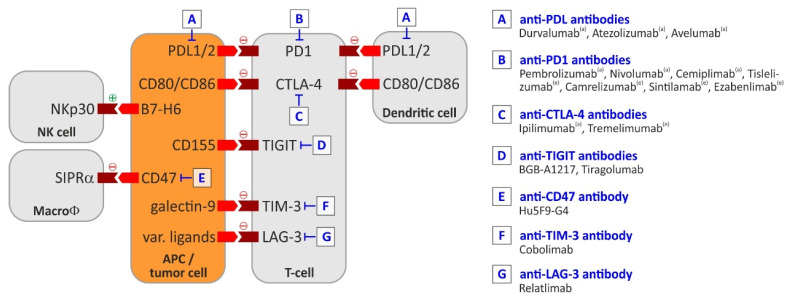
The most important immune checkpoints. Based on [16,17,18,19,20,21,22]. Abbreviations: APC, antigen-presenting cell; CTLA-4, cytotoxic T lymphocyte protein 4; LAG-3, lymphocyte activation gene 3 protein; PD1, programmed cell death protein 1; PDL1/2, programmed cell death protein ligand 1/2; TIGIT, T cell immunoreceptor with immunoglobulin and ITIM domains; TIM-3, T cell immunoglobulin and mucin-domain containing.

In the subsequent sections, this review summarizes the use of immunomodulatory agents for treatment of HCC–in particular, immune checkpoint inhibitors (ICIs). This subset of membrane-bound molecules fine-tune the immune response by preventing continuous T cell effector function after prior stimulation of antigen-specific T cells thus serving an immunosuppressive function to prevent uncontrolled T cell responses [16]. In the context of human cancer therapy, the currently most studied ICIs are PD1 (programmed cell death protein 1), CTLA-4 (cytotoxic T lymphocyte protein 4), LAG-3 (lymphocyte activation gene 3 protein), and, TIM-3 (T cell immunoglobulin and mucin-domain containing)–for an overview including the respective ligands, see also Figure 2.

As discussed recently [23,24], HCC represents a tumor which could be especially attractive for using immunomodulatory drugs because: (i) the liver itself covers important immune functions by filtering infectious agents from the blood flow or from the gastrointestinal system and, therefore, is permanently exposed to various antigens requiring a certain immune tolerability, and, (ii) HCC arises from typical inflammatory conditions (cirrhosis, hepatitis, see above) and can thus be considered an inflammation-related cancer with potential immunogenicity. The currently poor prognosis of HCC patients at advanced stages requires continuous efforts in identifying specific and more effective anti-HCC approaches. Here, the status of immunomodulatory drugs for treatment of HCC is discussed with special emphasis on currently available clinical data, possible combinations with established systemic therapies and local-ablative techniques as well as an outlook on possible future strategies.

## 2. Immunological Based Therapies in HCC

### 2.1. Established/Approved Immunotherapeutics in HCC 

#### 2.1.1. Established/Approved Immunotherapeutics in HCC

Treatment options in advanced HCC (BCLC C) have evolved rapidly over the last 3 years. After the implementation of the tyrosine kinase inhibitor (TKI) sorafenib in 2005 for advanced HCC [25], it took more than 10 years until levantinib was able to show comparable efficacy and was approved for the treatment of HCC [26]. The established first-line treatment options opened the possibility for second-line studies. After having progressed during sorafenib, treatment with regorafenib and cabozantinib showed efficacy in phase-III studies [27,28] and extended the use of TKI in HCC. Further treatment options in second-line consist of the use of ramucirumab (IgG1 targeting the extracellular domain of VEGF receptor 2), the first monoclonal antibody that has been approved for the use in HCC treatment [29]. The effect of ramucirumab was limited to those patients with elevated AFP levels. With an AFP level of higher than 400 ng/mL, the first predictive biomarker was introduced to the treatment of HCC. All those treatment options were in the pre-immune checkpoint era and consisted of TKIs or monoclonal antibodies. 

Early phase II studies investigating single agent use of immune-checkpoint inhibitors showed encouraging results and let to the premature approval of pembrolizumab (target: PD-1). The results of the respective phase III studies (KEYNOTE-240 and CheckMate 459) were disappointing. KEYNOTE-240 evaluated the efficacy of pembrolizumab in second line compared to placebo. The primary endpoints, OS and PFS, were improved by the use of pembrolizumab but did not meet their pre-specified statistical significance [30]. The use of nivolumab (target: PD-1) compared to sorafenib in the first-line setting was investigated in the CheckMate-459 study. The primary endpoint OS was not significantly improved [31] but both studies showed a favorable safety profile proofing the feasibility and low toxicity of immune checkpoint inhibitors in advanced HCC (aHCC).

The combination of immunecheckpoint inhibitors with anti-angiogenic substances or TKI’s revealed surprisingly positive results. Within the ImBrave-150 study, atezolizumab (target: PD-L1) was combined with bevacizumab (target: VEGF) and compared against sorafenib in first-line treatment of aHCC [32]. With Hazard ratios of 0.59 and 0.58 respectively, both, PFS and OS were statistically and clinically significantly improved. The use of atezolizumab and bevacizumab has set the new standard for first-line treatment of aHCC and recent data confirmed these preliminary data with a mPFS of 6.8 months and and ORR of 27% vs. 4.3 months and 12%, respectively, for sorafenib [33]. Table 1 gives and overview of the approved treatment options in HCC.

Ongoing studies evaluate the efficacy of double immunecheckpoint inhibition using PD-L1 inhibition and CTLA4 inhibition. The NCT02519348 study has shown efficacy and tolerability for the combination of tremelimumab (target: CTLA-4) and durvalumab (target: PD-L1) [34].

#### 2.1.2. Therapies with Immunologic Component

Locoregional therapy strategies (including transarterial embolization (TAE), transarterial chemoembolization (TACE), transarterial radioembolization (TARE), and ablative therapies like radiofrequency or (RFA) and microwave ablation (MWA)) are now routinely used in the adjuvant and neoadjuvant treatment of hepatocellular carcinoma [35]. Besides local therapeutic effects on tumor shrinkage, tumor necrosis and local reparative processes in the liver, systemic effects are already recognized, although the clinical relevance of this inflammatory response is not fully understood until now. Nevertheless, the increasing immunotherapy options for HCC raise the question, how combination treatment strategies could improve local ablative techniques and, vice versa, how those invasive procedures could impact on immunotherapy approaches. Therefore, the following chapter will summarize the known findings in animal studies and in patients as already recently reviewed in detail [36].

The first ablative experiments were performed with a locoregional VX-2 rabbit model, which served to establish the ablative techniques for clinical beginners and to investigate experimentally the “therapeutic” effects [37]. The application of VX2 was criticized due to following reasons: (i) the used VX2 tumor, an anaplastic squamous cell carcinoma induced by papilloma virus is not and does not reflect the typical HCC morphological and molecular phenotype; (ii) genetically heterogeneity between VX2 tumor specimen and animal recipient raise the question of being an allograft, rather than an autograft-model overall [36]. Therefore, animal models with spontaneous HCC development by treatment with the toxin diethylnitrosamine or by woodchuck hepatitis virus infection should reflect more the real immunological in situ situation than the “classical” VX2 tumor model [38]. A meta-analysis revealed that carcinogen induced tumor models showed the best correlation with clinical responses [39].

How does necrosis induce unspecific or even specific inflammatory response in these experimental in vitro and in vivo settings? Interestingly, while apoptosis, but not necrosis, was linked to the inflammatory reaction in vitro [40], the in vivo situation of the necrosis-inflammation-axis is quite complex, since immunogenic and non-immunogenic cell death is involved in this process [41]. Our own experiments with RFA in the VX2 model revealed that the local tumor control was paralleled by a local and systemic inflammatory reaction of activated T-cells [42]. The presented tumor antigens, released by tumor ablative techniques, could induce a localized immune response and activate a heterogeneous systematic immune response via antigen presenting cells like dendritic cells [43,44]. Additionally, combination of tumor ablation with checkpoint inhibitors like anti-CTLA4 could enhance anti-tumor immunity in vivo, too [45,46]. Consequently, the additional application of CpGs could improve this effect [47]. 

**Figure 3:**
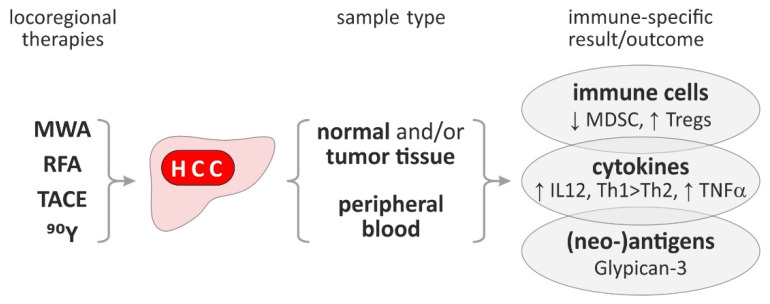
Overview of known immune effects of locoregional therapies for HCC. The arrows indicate the up- or downregulation of the observed immune effects. Based on [48,49,50,51,52,53,54,55,56]. Abbreviations: ^90^Y, XYZ; HCC, hepatocellular carcinoma; IL, interleukin; MDSC, myeloid-derived suppressor cells; MWA, microwave ablation; RFA, radiofrequency ablation; TACE, transarterial chemoembolisation; TNF, tumor necrosis factor; Treg, regulatory T cell.

Effects on the immune response were clinically investigated in different patients’ cohorts with HCC treated with different locoregional therapies like MWA, RFA, TACE or radioembolization with Y90 alone or in combination (as summarized in Figure 3). One major concern is linked to the fact, that the immune response is mostly analyzed in peripheral blood and not in the primary targeted liver tissue, limiting essentially the impact of such investigations. Furthermore, the immune outcome parameters are not strictly the same ranging from immune cells and cytokines to tumor-associated antigens. Lastly, transfer experiments of such “stimulated” immune cells and their cytokine and tumor-associated antigen counterparts are missing as proof of principle. Nevertheless, major findings of immune responses after locoregional treatment strategies of HCC are described in brief:

(1) MWA induces T-cell activation and IL-12 release [48,49].
(2) The RFA associated T cell response is specific to thermally ablated HCC extracts [50] and is also specific for tumor-associated antigens [51]. Furthermore, patients receiving RFA showed reduced frequency of myeloid-derived suppressor cells, which inversely correlates with tumor progression or relapse [52]. Treatment with RFA or TACE induces glypican-3 peptide specific cytotoxic T-lymphocytes compared to surgical resection which is a very interesting target for typical Glypican-3 overexpressing HCCs [53].
(3) Treatment with TACE leads to a change in inflammatory cytokine towards a Th2 profile [54] and an enhancement of CD4+CD25+ regulatory T cells [55].
(4) Radioembolization with Y90 leads to an increase in TNFA on CD4 and CD8 cells paralleled by an enhancement of antigen-presenting cells [56].

Finally, ongoing clinical trials investigated the combination of immune checkpoint inhibitors and locoregional ablative therapeutic strategies: Greten et al. initiated a clinical trial with 39 HCC patients who progressed after sorafinib therapy with a locoregional therapy after tremelimumab treatment [36] and confirmed the median overall survival of 10.9 months with a one complete and seven partial response as seen in an earlier study [57]. The additional molecular analysis of the peripheral blood of these treated patients revealed an increase of the PD1 expression on CD4+ and CD8+ T-cells.

Searching at the clinical trial registry (https://www.clinicaltrials.gov/ lastly accessed on 15 February 2021) with the term “HCC” for the disease input box and “immunotherapy and locoregional therapy” for other terms input box (last updated 3 February 2021) the database query indicates only six recruiting clinical trials (see Table 2). 

Due to the low number of studies and the heterogeneous study designs (different locoregional interventions, different combination partners), a more structured analysis of these strategies is needed in the future.

Taken together, there is evidence that tumor destruction via apoptosis and necrosis could induce a local immune response via activation of T cells and dendritic cells and via suppression of regulatory T cells and of myeloid-derived suppressor cells. This is associated with a change of inflammatory cytokines, whereby specific agonist like CpGs or antagonists like anti-CTL4 could enhance the anti-tumor immunity.

Under these circumstances, the clinical efficacy of immune modulation via checkpoint inhibitors is essentially influenced by the baseline immune response and by triggering pre-existing immunity, leading to the concept of “hot” and “cold” tumors on the basis of level and spatial distribution of CD3+ and CD8+ T cell infiltration into the tumor [58,59]. The already mentioned response rate of e.g., atezolizumab and bevacizumab in HCC is mostly comparable to a rate of “hot” HCC of about 20–30% [60,61]. Although this is in line with results found in many other cancers, it is surprising for HCC since the liver plays a central role in human immune regulation via the complex interaction of sinusoidal endothelial cells and resident macrophages (Kupffer cells) with NK cells and different CD4+/CD8+ T cell subsets and many HCCs develop on the basis of an underlying chronic inflammatory process [62,63]. As recently discussed elsewhere, the main issue to overcome the limitations of immunotherapy (alone or in combination) is to include the specific immunogenicity of tumor cells in relation to immune escape mechanisms in HCC [60]. Possible new treatment strategies for “cold” HCC could be based on intensive immune priming (e.g., vaccines, adoptive cell therapy or oncolytic approaches) and modulation (e.g., classical radiotherapy, chemotherapy and targeted therapy) to essentially enhance response to checkpoint inhibitors [58] as also addressed in the following sections.

### 2.2. Future Options of HCC Linked Immunmodulation

As shown in Figure 2, currently approved immune checkpoint inhibitors reactivate T cells by modulating the CTLA-4 or PD-1/PD-L1 signaling pathways. Interestingly, also other immunosuppressive molecules like TIM3 or LAG3 were found to be significantly increased in tumor-associated antigen specific HCC infiltrating CD8^+^ lymphocytes and may represent valuable targets for novel therapeutic approaches alone or in combination [64]. The general importance of T cell mediated anti-tumor effects has also been confirmed by an increasing number of CAR T approaches in HCC, usually targeting glypican 3 or AFP [65]. A recent report also demonstrated that hepatic FasL^+^CD11b^+^F4/80^+^ monocyte-derived macrophages can siphon activated CD8^+^ T cells and contribute to limited efficacy of immunotherapy [66]. In this section we will therefore expand the view also to myeloid cells like macrophages NK cells as well as the above-mentioned T cell checkpoints TIM-3, LAG-3 and TIGIT which have not yet been targeted in HCC.

#### 2.2.1. TIM-3

T cell immunoglobulin and mucin domain 3 (TIM-3), alias hepatitis A virus cellular receptor 2 (HAVCR2)) is an immunosuppressive surface molecule that is expressed on T cells, dendritic cells, NK cells, macrophages and also on HCC cells [67]. It is commonly co-expressed with other immune checkpoint receptors like PD-1. Activation of TIM3 leads to immune exhaustion of CD8^+^ T cells and its expression on CD4^+^ regulatory T cells (T_reg_) is associated with advanced tumor stage [68]. On macrophages, TIM-3 can stimulate the M2 polarization and promote tumor growth by increasing IL-6 secretion [69]. Not surprisingly, TIM-3 expression has thus been correlated to poor prognosis in various human cancers, including HCC [70,71,72]. Four ligands binding to TIM-3 have so far been identified: Galectin-9, phosphatidylserine, high-mobility group protein B1 (HGMB1) and carcinoembryonic antigen-related cell adhesion molecule-1 (CEACAM-1) [73]. Galectin-9 is produced by numerous cells types, including B and T cells, macrophages, and dendritic cells but also by epithelial cells, cancer cells and fibroblasts. In HCC, opposing effects of Galectin-9 have been described that are not well understood so far: while it is able to induce apoptosis in in vitro and in in vivo HCC models [74], it contributes to the immune exhaustion in HBV-associated HCC in patients and is a predictor for poor prognosis [72]. Interestingly, high levels of Galectin-9 have also been linked to advanced stages of liver fibrosis and cirrhosis in patients, underlining the connection between chronic inflammatory liver damage, fibrosis and HCC [75].

Several inhibitors for TIM-3 signaling have been developed (Table 3) [68,73]. While several compounds investigate TIM-3 blockade in various solid tumors, only one investigator sponsored study is specifically looking into HCC. Here, the anti-TIM-3 IgG4 antibody cobolimab is used in combination with the anti-PD1 antibody dostarlimab (both manufactured by Tesaro/GSK) in adult patients with BCLC stage B or C HCC and no prior systemic therapy. The study is ongoing and no interim data have been reported so far.

#### 2.2.2. LAG-3

The lymphocyte activation gene 3 protein (LAG-3; CD223) is another strong suppressor of T cell function. It is expressed on tumor infiltrating lymphocytes (CD4^+^ and CD8^+^ T cells), T_reg_, NKT cells. B cells, NK cells, plasmacytoid dendritic cells (pDCs) and on tumor associated macrophages (TAMs) [76]. It regulates the immune response by inhibiting the proliferation and activation of T cells, by inducing T_reg_ and by blocking T cell activation from antigen presenting cells (APCs) [77]. LAG-3 is commonly co-expressed with PD-1 in T cell exhausted cancers and contributes to resistance to immune checkpoint inhibitor therapy [78,79,80]. For LAG-3, too, four ligands have been identified today: major histocompatibility complex class II proteins (MHC-II) [81], liver sinusoidal endothelial cell lectin (LSECtin) [82], Galectin-3 [83] and fibrinogen-like protein 1 (FGL-1) [84]. All ligands are of relevance for HCC formation: while MHC-II is expressed on activated APCs (Kupffer cells), the other ligands can be expressed by hepatocytes or sinusoidal endothelial cells which also play a role in chronic liver damage, fibrotic remodeling, angiogenesis and tumor formation [85,86,87,88,89]. LAG-3 expression has therefore also been associated to poor prognosis in various human cancers including HCC [90,91]. 

Preclinical data indicated a strong anti-tumor efficacy of LAG-3 antagonists, esp. when combined with anti-PD-1 agents [92,93,94,95]. Thus, about 15 large-molecule antagonists against LAG-3 (either mono- or bispecific against PD-1) are currently investigated preclinically or in early clinical studies (recently reviewed by Lecocq et al. [76]). Yet, single agent activity if those compounds was only limited and most trials now combine anti-LAG-3 with anti-PD-1 approaches. Currently, five studies investigating such approaches in HCC are listed at clinicaltrials.gov (Table 4). So far, only the Phase 1 study for INCAGN02385 (NCT03538028) is completed and enrolled a total of 22 patients across multiple solid tumor indications, including HCC, but no data was reported so far. Specific studies for HCC are only conducted with the IgG4 anti-LAG3 antibody relatlimab (BMS-986016) in combination with nivolumab in either resectable (NCT04658147) or in immunotherapy naïve patients after failure of tyrosine kinase inhibitors (NCT04567615). 

#### 2.2.3. TIGIT

The T cell immunoreceptor with immunoglobulin and ITIM domains (TIGIT) is expressed on activated NK and T cells, including CD4+ and CD8+ T cells, as well as T_reg_ and T helper cell populations under resting conditions to exert an immunosuppressive condition [96]. CD155 was identified as the main ligand, mainly expressed on DCs, macrophages, B and T cells. CD112 (Nectin-2) and CD113 (Nectin-3) bind to TIGIT with lower affinity and all three ligands can also be detected in the liver. TIGIT was found to be upregulated in patients with advanced fibrosis [97] and in chronic viral hepatitis leading to HCC [98]. In preclinical HCC models, TIGIT contributed to immunosuppressive effects and potentially resistance to PD-1 treatment [99,100]. In clinical samples, TIGIT expression increased with tumor dedifferentiation and with higher AFP expression [101].

Several monoclonal anti-TIGIT antibodies, usually IgG1 subtypes, are currently undergoing early clinical testing (recently reviewed by Harjunpaa and Guillerey [96]). Most compounds are tested in combination with anti-PD-1 or anti-PD-L1 antibodies but no study is specifically investigating HCC yet. Recently, tiragolumab in combination with atezolizumab received FDA breakthrough therapy designation for the first-line treatment of metastatic non-small cell lung cancer with high PD-L1 expression and no mutations in EGFR or ALK [102]. Further studies that also investigate HCC are expected. For other compounds, e.g., vibostolimab (MK-7684), etigilimab (OMP-313M32), domvanalimab (AB-154), BMS-986207, ASP8374 or BGB-A1217 are currently in Phase 1 studies in various solid tumors with a focus on NSCLC.

#### 2.2.4. B7-H6

The B7 receptor family (alias natural cytotoxicity triggering receptor 3 or NCR3, Ligand 1) represents co-receptors to e.g., CTLA-4 or PD-1 [103]. B7-H6 is a ligand to the activating receptor NKp30 on NK cells and thus contributes to their activation [104]. B7-H6 mediated activation of NK cells leads to cytokine release (IFN-g) and enhanced cytotoxicity. Besides immunological effects, B7-H6 does also regulate intracellular signaling pathways, esp. STAT3 signaling, which are associated with apoptosis inhibition and induction of cell proliferation and therefore has a dual role in cancer cell growth [105,106]. 

While B7-H6 is usually not expressed in normal tissues, it is commonly found in different human cancers like small cell lung cancer [107], esophageal squamous cell carcinoma [108], gliomas [109], ovarian cancer [110] or HCC [106,111], where it is associated with poorer outcome. Unfortunately, no agents modulating B7-H6 signaling on tumor or NK cells are currently available [112]. 

#### 2.2.5. CD47-SIRPa

CD47 is broadly expressed on normal cells, including erythrocytes. It belongs to the immunoglobulin superfamily and displays a “don’t eat me”-signal to macrophages and other phagocytes. Binding of CD47 to its receptor signal regulatory protein a (SIRPa) on macrophages inhibits phagocytosis activation and can contribute to tumor formation [113,114]. CD47 is therefore overexpressed on various hematologic and solid tumors to evade the cellular immune response, including HCC where it is also associated to poorer outcome [21]. Consequently, blocking CD47-signaling inhibited growth of HCC models and restored sensitivity to chemotherapy [115]. 

Activation of CD47 on tumor cells can also lead to caspase-independent cell death induction, although the exact molecular mechanisms are still not completely understood [116]. Therapeutic approaches currently focus on inhibiting the CD47-SIRPa binding to activate phagocytosis of cancer cells and several small and large molecule inhibitors are undergoing clinical investigations. Small molecule inhibitors are currently in preclinical stage only and have been recently reviewed elsewhere [117]. Table 5 gives an overview of large molecule CD47 inhibitors in early clinical trials. None of these agents is specifically investigated in HCC.

Recently, a Phase 1 study with the bi-functional SIRPa-Fc-CD40L antibody SL-172154 was initiated (NCT04406623). This agent targets CD47 on tumors and CD40 on antigen presenting cells to enhance antigen presentation to T cells and to induce tumor cell killing.

#### 2.2.6. Additional in-Silico-Analysis of HCC Linked Immunmodulation via TUMOR Immune Estimation Resource (TIMER) 

We performed an additional in silico analysis of TIM3, LAG3, TIGIT, B7-H6 and CD47-SIRPa to explore the correlation of these markers of immunomodulation in situ by using the online platform TIMER, which is based on 10,897 samples across 32 cancer types from The Cancer Genome Atlas (TCGA) [118]. This included 363 primary HCC samples with mainly male patient population (66%) of caucasian ethnicity (60%) showing mostly a moderate differentiation (50%) and a relative homogenous UICC-stage distribution (Stage I 39%, II, 22%, III 31% and IV 3%. missing 6%) as already published [61].

**Figure 4:**
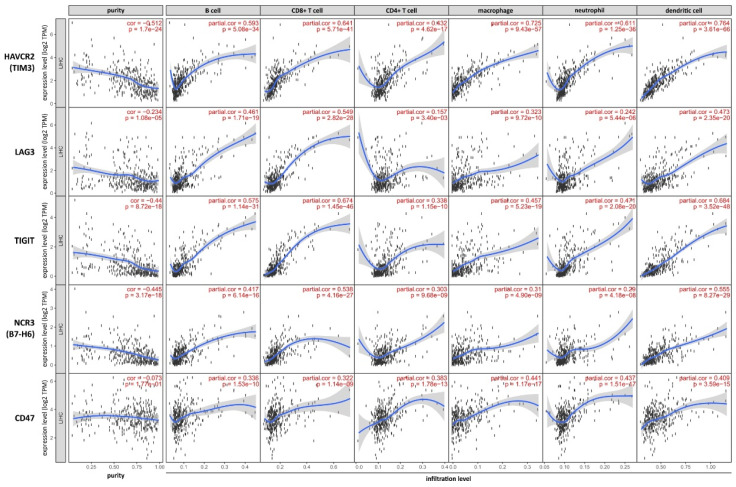
In silico analysis using TIMER with the gene module for markers of immune modulation TIM3, LAG3, TIGIT, B7-H6 and CD47-SIRPa in HCC.

We focused on the gene module of TIMER to investigate the correlation with the tumor purity and the six tumor infiltration subsets of B cells, CD8+ T cells, CD4+ T cells, macrophages, neutrophils and dendritic cells in HCC as presented in Figure 4.

Overall, all markers of immunomodulation showed a negative correlation with the tumor purity indicating that all markers are more found at the tumor border than in the tumor center. Furthermore, all markers of immunomodulation were positively associated with B cells, CD8+ T cells, CD4+ T cells, macrophages, neutrophils and dendritic cells in HCC, with a partial correlation factor reaching up to 0.725/0.764 for TIM3 and macrophages/dendritic cell. This indicates a very strong association with tumor infiltrating immune subsets in HCC, especially with antigen presenting cells. 

This in silico analysis revealed two major patterns of correlation in dependency of infiltration density of tumor infiltrating immune cells: immunomodulators like TIM3 and TIGIT showed parallel increasing expression, while the immunomodulators LAG3, B7-H6 and CD47 displayed a heterogeneous expression pattern compared to the density of tumor cells. Taken together, the in silico analysis indicates that the density of tumor infiltrating immune cells like B cells, CD8+ T cells, CD4+ T cells, macrophages, neutrophils and dendritic cells is mostly paralled by the expression of selective markers of immunmodulation. Therefore, the tumor compartments as well as the specific subsets of immune cells, too, must be integrated in the evaluation as biomarkers for consecutive immune checkpoint therapy in HCC.

## 3. Conclusions

In summary, the growing understanding and knowledge of carcinogenesis, diagnosis and possible treatment strategies for patients with HCC did so far not essentially improve the outcome of patients suffering from HCC, with 5-year survival rates of only about 8% and high tumor recurrence rates after resection [119]. Consequently, investigating alternative therapeutic strategies for HCC is an urgent necessity [65]. 

The development of immune checkpoint inhibitors like anti-CTLA4 (ipilimumab, tremelimumab) or anti-PD-1/PD-L1 (nivolumab, pembrolizumab, tislelizumab, camrelizumab, atezolizumab, durvalumab, avelumab) antibodies has dramatically revolutionized the field of clinical oncology leading to impressing treatment responses in different human cancer types.

Results from recent clinical trials combining a checkpoint inhibitor with either multi-tyrosine kinase inhibitors or angiogenesis inhibitors confirmed that HCC is an inflamed (“hot”) tumor. The combination of atezolizumab and bevacizumab received approval for 1st line therapy. Still, the objective response rates of these combinations barely exceed 25 to 30%, indicate that further therapy optimization is needed.

Basic scientific investigations of HCC in vitro and in vivo, especially after locoregional treatment, created evidence that therapeutic induction of apoptosis and/or necrosis leads to significant changes of the immune cell repertoire and of immune modulating cytokines in the liver and systemically in the whole human body/system. Consequently, the idea was born to combine immune checkpoint inhibitors and locoregional treatments of HCC to potentiate the local and systemic immune response. The first results of such clinical trials gave encouraging signs on clinical endpoints like overall survival and response rate. Nevertheless, this approach of immunotherapy in combination with locoregional therapeutic approaches raised many “best” questions on the treatment modality, timing and biomarkers which must be solved in the future: What treatment modality (locoregional treatment and delivery mode of immunotherapy) is the best choice for maximal immune triggering? Which and what timing of combination is the best for deep and long-time tumor response of the HCC? Which biomarker is the best to indicate the most suitable checkpoint inhibitor? 

Taken all together, recent developments of immunomodulatory treatment strategies alone or in combination will essentially change our therapeutic options for the HCC in the future. 

## Figures and Tables

**Table 1:** Approved substances in the treatment of aHCC.

Substance	Year of Approval	Study	Comments and Primary Endpoint
**First-Line Options**			
Sorafenib	2005	SHARP	OS vs. placebo: 10.7 mo vs. 7.9 mo; (HR 0.69)
Levantinib	2018	REFLECT	Non inferiority to sorafenib OS: 13.6 mo vs. 12.3 mo (HR 0.92)
Atezolizumab + Bevacizumab	2020	ImBrave-150	OS vs. sorafenib OS: not reached vs. 13.2 mo (HR 0.58)
**Second-Line Options**			
Regorafenib	2017	RESORCE	After sorafenib first-line vs. BSC OS: 10.6 mo vs. 7.8 mo (HR 0.63)
Cabozantinib	2019	CELESTIAL	After sorafenib first-line vs. BSC OS: 10.2 mo vs. 8.0 mo (HR 0.76)
Ramucirumab	2019	REACH-2	After sorafenib first-line vs. BSC in patients with AFP >400 ng/mL OS: 8.5 mo vs. 7.3 mo (HR 0.71)

AFP: alpha fetoprotein; aHCC: advanced hepatocellular carcinoma; BSC: best supportive care; HR: hazard ratio; OS: overall survival; mo: months.

**Table 2:** Ongoing studies investigating the combination of locoregional therapies and immunotherapy.

Start Date	NCT	Title	Local Interventions	Immuno- Modulator	Phase
01/2020	NCT04220944	Combined locoregional treatment with immunotherapy for unresectable HCC.	MWA/TACE	Sintilimab	1
05/2019	NCT03753659	IMMULAB-immunotherapy with pembrolizumab in combination with local ablation in hepatocellular carcinoma (HCC)	RFA, MWA, Brachytherapy, TACE	Pembrolizumab	2
11/2019	NCT04273100	PD-1 monoclonal antibody, lenvatinib and TACE in the treatment of HCC	TACE	PD-1 mAb and lenvatinib	2
09/2020	NCT04518852	TACE, Sorafenib and PD-1 monoclonal antibody in the treatment of HCC	TACE	sorafenib and PD-1 mAb	2
05/2019	NCT03867084	dafety and efficacy of pembrolizumab (MK-3475) versus placebo as adjuvant therapy in participants with hepatocellular carcinoma (HCC) and complete radiological response after surgical resection or local ablation (MK-3475-937/KEYNOTE-937)	Local ablation	Pembrolizumab	3
05/2019	NCT04268888	Nivolumab in combination with TACE/TAE for patients with intermediate stage HCC	TACE/TAE	Nivolumab	2/3

HCC: hepatocellular carcinoma; MWA: microwave ablation; RFA: radiofrequency ablation; TACE: trans-arterial chemo-embolization; TAE: trans-arterial embolization.

**Table 3:** TIM-3 inhibitors in clinical development.

Compound	Company	Status/Comment
BMS-986258	BMS	Phase 1 in solid tumors in combination with nivolumab
Cobolimab (TSR-022, GSK4069889)	Tesaro/GSK	Various Phase 1 studies ongoing +PD-1 in HCC (NCT03680508)
INCAGN02390	Incyte	Phase 1 in solid tumors
LY3321367	Eli Lilly	PD-1/TIM-3 bispecific Development stopped
RG7769 (RO7121661)	Roche	PD-1/TIM-3 bispecific Phase 1 in solid tumors
Sabatolimab (MBG 453)	Novartis	Only in hematologic malignancies
Sym023	Symphogen	Phase 1 in combination with PD-1 and/or LAG-3 antibodies

**Table 4:** LAG-3 inhibitors investigated in HCC.

Compound	Company	Combination	N	Phase	NCT
INCAGN02385	Incyte		22 (advanced solid tumors)	1	NCT03538028
Relatlimab	BMS	Nivolumab	20	1	NCT04658147
Relatlimab	BMS	Nivolumab	250	2	NCT04567615
SRF388	Surface Oncology		122 (advanced solid tumors, with n = 40 HCC expansion arm)	1	NCT04374877
XmAb^®^22841	Xencor	Pembrolizumab	242 (advanced solid tumors)	1	NCT03849469

**Table 5:** Anti-CD47 antibodies in early clinical development.

Compound	Company	Status/Comment
AK117	Akeso	Phase 1
ALX148	ALX Oncology	Phase 2 combinations
AO-176	Arch Oncology	Phase 1, combination with paclitaxel
CC-90002 (INBRX103)	Celgene	Phase 1
HX009	Hanxbio	Phase 1
IBI188	Innovent Biologics	Phase 1
IBI322	Innovent Biologics	Phase 1
IMC-002	ImmuneOncia Therapeutics	Phase 1
Magrolimab (Hu5F9-G4)	Gilead	Phase 3, received breakthrough therapy designation for MDS, Phase 1b combination studies in solid tumors
SGN-CD47M	Seattle Genetics	Terminated
SRF231	Surface Oncology	Phase 1 completed
ZL-1201	ZaiLab	Phase 1

## Data Availability

No new data were created or analyzed in this study. Data sharing is not applicable to this article.
